# ETHICAL DILEMMAS PRESENTED BY COGNITIVE IMPAIRMENT AND VULNERABLE ELDERS

**DOI:** 10.1093/geroni/igz038.888

**Published:** 2019-11-08

**Authors:** Elizabeth J Santos, Corey A Nichols-Hadeed

**Affiliations:** 1 University of Rochester School of Medicine & Dentistry, Rochester, New York, United States; 2 University of Rochester Medical Center, Rochester, New York, United States

## Abstract

Identification and assessment for cognitive impairment is a difficult task further complicated by the need to determine capacity. Issues related to cognitive impairment and capacity create ethical dilemmas potentially spanning all four ethical principles: autonomy, beneficence, non-malfeasance, and justice. This paper uses a case scenario to describe different types of cognitive impairment and demonstrate ethical issues that commonly arise when treating patients with cognitive impairment in the clinical setting. The authors also recognize the complexity of capacity as an issue that spans both the medical and legal fields and provides explanations and distinctions. The overall goal of this paper is to raise awareness of the impact of cognitive impairment on the vulnerability of older adults, describe the complex ethical issues that cognitive impairment and capacity raise and the importance of defining capacity in the context of the legal and medical fields.

